# Crohn's disease: An enigmatic variant with gastritis and ileal obstruction

**DOI:** 10.1002/ccr3.8371

**Published:** 2023-12-27

**Authors:** Samuel Amo‐Tachie, Fredrick Kojo Ocansey, Abena Durowaa Yeboah

**Affiliations:** ^1^ Department of Medicine and Therapeutics University of Ghana Medical School, University of Ghana Accra Ghana; ^2^ Department of Internal Medicine Cape Coast Teaching Hospital Cape Coast Ghana

**Keywords:** constipation, Crohn's disease, gastritis, intestinal obstruction

## Abstract

There are multiple atypical manifestations of Crohn's disease, which sometimes delay diagnosis or even more often result in complete misdiagnosis, especially in poorly equipped facilities. This is the case of an elderly woman with Crohn's disease who presented with gastritis and bowel obstruction. She had hitherto been wrongly managed for peptic ulcer disease and functional constipation based mainly on her symptoms. Her diagnosis was made only after years of failed symptomatic management. This case aims to highlight the uncommon and easily misdiagnosed gastroduodenal presentation of Crohn's disease, as well as clinical clues to correctly diagnosing the condition.

## Graphical abstract

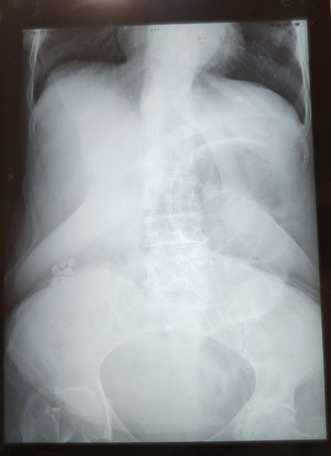

## 1 INTRODUCTION

Crohn's disease falls under the umbrella of inflammatory bowel disease and can involve any part of the gastrointestinal tract. It is many a time misdiagnosed or diagnosed late.^1^ This is even more so when health centers are not well equipped to appropriately investigate it, as is the case in many developing countries. Common presentations include abdominal pain, diarrhea, unintentional weight loss, and hematochezia. It is more prevalent in young adults.^2^ There are however enigmatic variants that do not present with these telltale symptoms. These include those that manifest with obstructive symptoms, dyspeptic symptoms as well as those with more extra‐intestinal than gastrointestinal symptoms such as arthritis, aphthous stomatitis, and uveitis.

## 2 CASE PRESENTATION

This is the case of a 78‐year‐old Ghanaian woman who was admitted on account of an acute exacerbation of Crohn's disease. She was diagnosed about a year prior to the index presentation through an investigation for incomplete intestinal obstruction. No treatment was started after the diagnosis, as she declined medical interventions. The index presentation was a week's history of severe generalized abdominal pain graded 9/10, colicky in her lower abdomen, and burning in her epigastric region. It was associated with constipation (hard, scanty, infrequent stools averaging once a week) and vomiting, which was non‐projectile, non‐bilious, and non‐bloody. The symptoms waxed and waned and were worse at night. She also reported having anorexia and unintentional weight loss, but no pain related to eating or fasting.

Antacids gave partial relief to the epigastric pain. There was no abdominal distension related to her symptoms, and her constipation was not absolute, as she was able to pass flatus. There was no alternation of the constipation with diarrhea, and there was no melena or hematochezia. Her other symptoms included borborygmi, lower back, and bilateral knee pain. She had hypertension well‐controlled on amlodipine. She had completed empirical triple therapy for peptic ulcer disease several years prior to this presentation but without resolution of her occasional dyspeptic symptoms. There was no history of abdominal surgeries, and she had never smoked or consumed alcohol.

Her physical examination showed a soft, non‐distended abdomen that moved with respiration. There was generalized tenderness, which was worst in her lower abdomen. There were no palpable masses, percussion notes were tympanitic and bowel sounds were high‐pitched and frequent. Both knees were mildly tender on passive joint movement and worse on the left, without warmth or swelling, and there was mild tenderness at her lower back. All other examination findings were normal.

Her renal and liver function tests were normal. She however had normocytic normochromic anemia of 10.6 g/dL on her complete blood count and low calcium and magnesium levels of 1.61 mmol/L, and 1.07 mmol/L respectively. An erect and supine abdominal x‐ray showed mildly dilated jejunum as well as gall stones. A contrast CT scan of the abdomen showed mild to moderate narrowing of the mid‐segment of the ileum with prestenotic dilatation of the ileum and jejunum. The dilated bowel measured 3.8–4.5 cm. There were no masses seen, and the large bowel was normal. Both ESR and CRP were high (30 mm/h and 147 mg/L, respectively). Stool RE was normal, and stool for occult blood and *Helicobacter pylori* antigen were negative.

She was then managed for acute exacerbation of Crohn's disease with partial ileal obstruction and gastroduodenal involvement. The noted Crohn's disease associations were cholelithiasis, arthritis of both knees and sacroiliitis. Her calculated Crohn's Disease Activity Index (CDAI) was 263, for moderate disease. She was hydrated with IV fluids, then put on hyoscine bromide for her crampy abdominal pains, IV omeprazole for her gastritis, SC enoxaparin for DVT prophylaxis, and syrup lactulose for her constipation. After her vomiting subsided, she was switched to oral medications.

Oral mesalazine 1 g 6 hourly, oral prednisolone 30 mg daily, and oral paracetamol 1 g 8 hourly were added to these medications. She had dietary interventions such as reducing meal fiber as well. She improved, had a significantly reduced frequency of flares, and was finally discharged after 5 days of admission.

## 3 DISCUSSION

Gastroduodenal Crohn's disease is not commonly symptomatic and is seen in only about 4% of cases.^3^ Its diagnosis is difficult because there are no specific or consistent pathological findings, as well as the high prevalence of comorbidity with *H. pylori* gastritis.^4^ Medical therapy is usually enough in most cases, except for those with gastric outlet obstructions that may require surgery.^5^ This patient had peptic ulcer‐like symptoms, which had been treated unsuccessfully in the past. That she was managed empirically for peptic ulcer disease for a long period because of the low availability of endoscopy in the Central Region of the country. Lack of these investigative facilities and low socioeconomic status result in the frequent use of mainly clinical impressions to make diagnoses in smaller facilities. Since dyspeptic symptoms are rare in Crohn's disease,^6^ they are unlikely to be the clinical clue to an accurate diagnosis, which unfortunately was the case in this patient for several years. Her age of presentation was also unusual, as Crohn's disease is usually diagnosed in young people.

Intestinal obstruction caused by the strictures of Crohn's disease widens the differential diagnoses for clinicians. These include intraluminal causes such as tumors, impacted feces, and foreign bodies; intramural causes such as intussusception; and extramural causes such as strangulated hernias, adhesions, and volvuli.^7^, ^8^, ^9^ Most of these are purely surgical causes. It is no surprise that this patient was managed by the general surgery team on her first admission. It is however quite unusual that she had never experienced diarrhea at any point in the course of the disease because it is expected that before the strictures form, the preceding inflammation would cause irritative diarrheal symptoms.^10^

Intestinal strictures form part of the natural history of Crohn's disease and will occur in up to 70% of patients over a 10‐year period.^11^ The strictures are classified as inflammatory, fibrotic, primary, or anastomotic.^12^ Inflammatory strictures result from edema of the intestinal lining, while fibrotic ones are caused by chronic inflammation that results in the accumulation of extracellular matrix and hyperproliferation of smooth muscle cells.^13^ There are also mixed variants that have both mechanisms at play at the same time. Primary strictures are those that follow the natural history of the disease, and anastomotic ones are those resulting from surgical intervention, such as resection and anastomosis. This woman had a primary stricture that was most likely mixed inflammatory and fibrotic because of the waxing and waning nature of her baseline constipation associated with the flares. Fibrotic strictures tend to produce a more constant type of obstruction, while inflammatory ones occur in waves.

**FIGURE 1:**
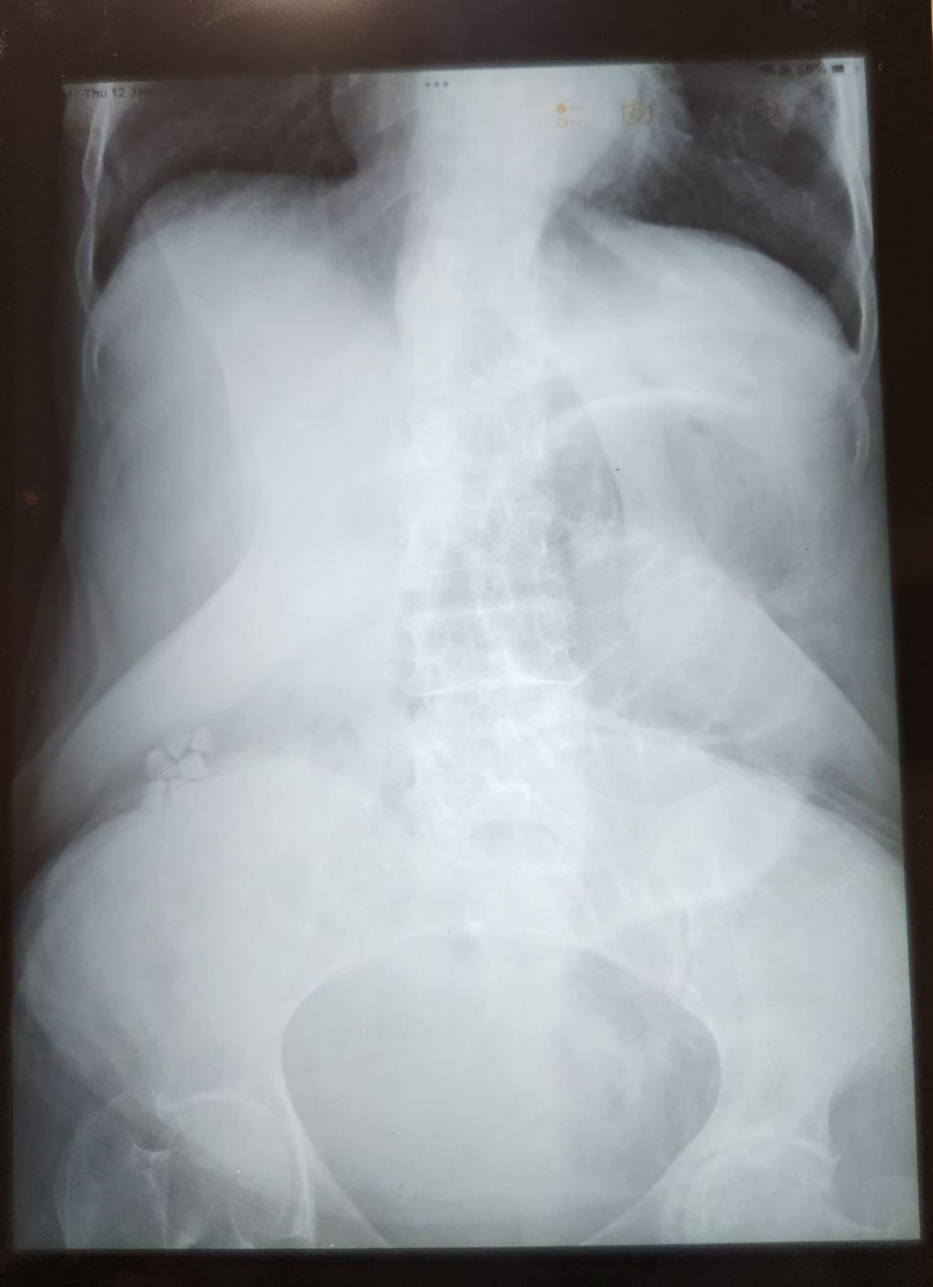
Supine abdominal x‐ray showing gallstones and mild jejunal dilatation.

**FIGURE 2:**
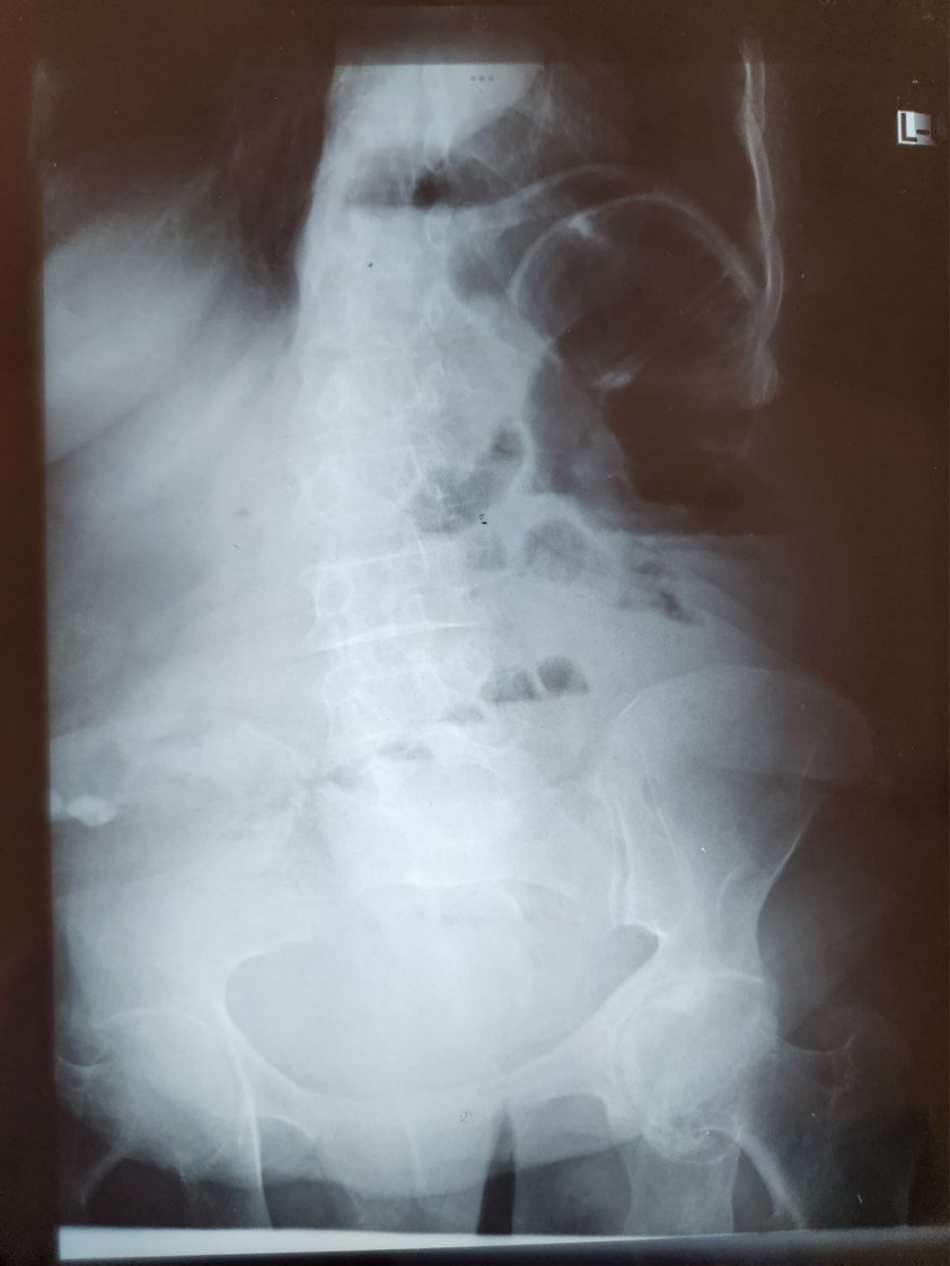
Erect abdominal x‐ray.

Medical management was her mainstay because of her advanced age and moderate disease severity (CDAI 263). Dietary modifications such as reducing fiber in the diet are necessary for reducing obstructive symptoms by reducing stool bulk to enable them to bypass strictures.^12^ Micronutrient supplementation may also be needed in cases where deficiencies such as hypocalcemia, hypomagnesemia, and vitamin D are a concern due to malabsorption, as was the case in this patient. The deficiencies also result from anorexia caused by abdominal pain (gastritis in this patient) and circulating inflammatory cytokines.^14^ Malabsorption is also the reason for the formation of gallstones, as in this patient (Figures 1 and 2). The gallstones form as a result of failed reabsorption of bile acid and hence failed enterohepatic circulation, which in turn increases the concentration of cholesterol in the biliary system, precipitating the formation of cholesterol stones.^15^ It is usually asymptomatic and an incidental finding on radiography.

Starting the patient on prednisolone and mesalazine resulted in a decline in symptoms by the 5th day of admission. 5‐aminosalicylic acid derivatives such as mesalazine provide symptomatic relief for patients and remain the first‐line drugs for mild to moderate disease.^16^ Lactulose also improved her constipation by enabling her to pass soft stools daily. Surgery is indicated in situations such as significant prestenotic dilatation or the presence of a fistula,^11^ none of which was present in this case.

## 4 CONCLUSION

Bowel obstruction from strictures in Crohn's disease is a common complication that may not always be preceded by diarrhea in the early stages, as was in this case. Gastroduodenal Crohn's disease is uncommon but should be considered in all patients being worked up for Crohn's disease who have dyspeptic symptoms. Negative *H. pylori* antigen tests or persistence of symptoms after eradication of *H. pylori* should increase suspicion of gastroduodenal Crohn's disease. These clinical clues are necessary not only for poorly equipped facilities but also for those that have all the investigative capacity in order to prioritize investigations and intervene as soon as possible.

## AUTHOR CONTRIBUTIONS

**Samuel Amo‐Tachie:** Conceptualization; data curation; formal analysis; methodology; project administration; resources; supervision; visualization; writing – original draft; writing – review and editing. **Fredrick Kojo Ocansey:** Visualization. **Abena Durowaa Yeboah:** Visualization.

## CONFLICT OF INTEREST STATEMENT

None declared.

## FUNDING INFORMATION

The authors have no funding or financial relationships to disclose.

## CONSENT

Written informed consent was obtained from the patient to publish this report in accordance with the journal's patient consent policy.

## Data Availability

The data that support the findings of this study are available on request from the corresponding author [SAT].
